# Intestinal acetate and butyrate availability is associated with glucose metabolism in healthy individuals

**DOI:** 10.1016/j.isci.2023.108478

**Published:** 2023-11-16

**Authors:** Madelief Wijdeveld, Anouk Schrantee, Anouk Hagemeijer, Aart J. Nederveen, Torsten P.M. Scheithauer, Johannes H.M. Levels, Andrei Prodan, Willem M. de Vos, Max Nieuwdorp, Richard G. Ijzerman

**Affiliations:** 1 Department of Internal and Experimental Vascular Medicine, Amsterdam University Medical Centers, location AMC, 1105 AZ Amsterdam, Noord-Holland, the Netherlands; 2 Department of Radiology and Nuclear Medicine, Amsterdam University Medical Center, location AMC, 1105 AZ Amsterdam, Noord-Holland, the Netherlands; 3 Department of Endocrinology, Amsterdam University Medical Centers, location VUMC, 1081 HV Amsterdam, Noord-Holland, the Netherlands; 4 Department of Microbiology, Wageningen University, 6708 PB Wageningen, Gelderland, the Netherlands; 5 Human Microbiome Research Program, Faculty of Medicine, University of Helsinki, 00100 Helsinki, Uusimaa, Finland

**Keywords:** Human metabolism, Microbiome

## Abstract

Animal studies suggest that short-chain fatty acids acetate and butyrate are key players in the gut-brain axis and may affect insulin sensitivity. We investigated the association of intestinal acetate and butyrate availability (measured by butyryl-coenzyme A transferase (ButCoA) gene amount) with insulin sensitivity and secretion in healthy subjects from the HELIUS cohort study from the highest 15% (N = 30) and the lowest 15% (N = 30) intestinal ButCoA gene amount. The groups did not differ in insulin sensitivity or secretion. However, the high ButCoA group showed lower glucose and insulin peaks during the first 60 min after a meal and a higher nadir during the second 60 min (p < 0.01), suggesting delayed glucose adsorption from the small intestine. Our data suggest that chronically increased acetate and butyrate availability may improve glucose metabolism by delaying gastric emptying and intestinal adsorption. Future studies should further investigate the effect of acetate and butyrate interventions.

## Highlights

• High levels of ButCoA signify high intestinal acetate and butyrate availability
• High intestinal ButCoA associates to lower postprandial glucose and insulin levels
• High ButCoA associates to a lower CNS response to anticipation of palatable food
• High ButCoA did not associate to insulin sensitivity or postprandial glucose iAUC

## Graphical abstract

**Figure:**
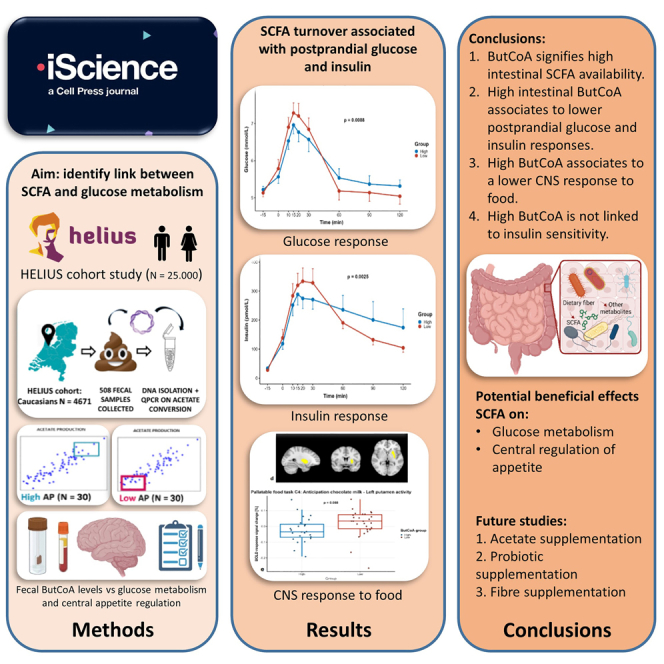

## Introduction

Obesity is a complex condition caused by multiple factors including food abundance, systematic overeating, and a sedentary lifestyle. Disturbed appetite regulation is a key factor underlying obesity, mainly caused by central desensitization to food intake,^1^ which is in turn influenced by a variety of factors including long-term leptin signaling, nutrient-induced feedback from the gastrointestinal tract (ghrelin, GLP-1, GIP, and other hormones), and the resulting neurotransmitter signaling. However, recent evidence also suggests a contributing role for the gut microbiome in pathophysiological pathways that lead to overeating, obesity, insulin resistance, and type 2 diabetes mellitus.^2^

In this regard, short-chain fatty acids (SCFAs) produced by fermentation of dietary fiber in the gut are suggested key players that may mediate the interaction of gut microbiota with both the host glucose metabolism and central nervous system (CNS).^3^ Acetate, the most abundant SCFA, has been suggested to interact with multiple bodily processes, affecting appetite regulation and postprandial glucose and insulin responses. In rodents, acetate binds to peripheral free fatty acid receptors (FFARs) on pancreatic islets and hepatocytes and hence modulates both glucose metabolism and insulin secretion.^4^ Additionally, acetate may function as a beneficial substrate improving both glucose levels and insulin secretion as well as central appetite regulation in rodents.^5^ In mice, increased intestinal acetate concentrations have been linked to an altered expression of regulatory neuropeptides favoring appetite suppression.^5^ Oral butyrate has also decreased food intake and suppressed orexigenic neurons expressing hypothalamic neuropeptide Y.^6^ Furthermore, dietary supplementation of butyrate can prevent and treat diet-induced insulin resistance in dietary obese C57BL/6J mice^7^ and in 5/6th nephrectomy rats.^8^ Nevertheless, these animal findings are still controversial.^9^ In humans, acetate is able to pass the blood-brain barrier^10^ and there is some evidence that acetate (administered by dietary vinegar supplementation) is associated with acute beneficial effects on appetite and insulin sensitivity.^11^ However, the association of chronically elevated intestinal SCFA levels with glucose levels, insulin sensitivity and secretion, and central regulation of food intake has not yet been investigated. In a preliminary study, oral butyrate supplementation in humans increased insulin sensitivity in a subgroup of lean but not obese individuals.^12^ If increased acetate and butyrate indeed prove to be beneficial for human metabolic health and appetite regulation, this would offer opportunities for various non-invasive intervention strategies to improve metabolic health.

Between individuals, there is a large variation in intestinal acetate and butyrate availability.^13^ We thus performed an observational study to examine the association of long-term intestinal microbial acetate and butyrate availability with insulin sensitivity, insulin secretion, and central appetite regulation. To this end, we selected individuals from the highest 15% (N = 30) or the lowest 15% (N = 30) acetate and butyrate availability (estimated as the fecal concentration of the butyryl-coenzyme A (CoA)-transferase (ButCoA) gene^14^) from the large HELIUS cohort.^15^ The link between ButCoA and intestinal SCFA turnover is supported by multiple lines of evidence. First, the study of Diez-Gonzalez et al.^14^ showing a clear positive relation between ButCoA activity and acetate in the butyrate producer *B.* *fibrisolvens*. Second, many abundant gut butyrogens like Faecalibacterium, Anaerostipes, and Anaerobutyricum spp. incorporate exogenous acetate to produce butyrate via ButCoA in an ATP generating reaction, supporting the relation between ButCoA abundance and acetate and the direct relation of ButCoA abundance and butyrate.^16^^,^^17^ Third, early textbooks that may have not received great attention already described the role of ButCoA in Clostridium subspecies.^18^ Glucose and insulin responses during a standardized mixed meal test (SMMT) were measured, as well as ghrelin and GLP-1. In addition, we determined neural activity in reward and satiety related areas in response to visual food cues and the anticipation and receipt of palatable food, by means of functional magnetic resonance imaging.

## Results

### Clinical characteristics

A total of 30 subjects with high ButCoA gene abundance (mean 367.07% ± 205.97% ButCoA copies per 16S rRNA gene copy) and 30 subjects with low ButCoA gene abundance (mean 7.17% ± 7.93% ButCoA copies per 16S rRNA gene copy) were included in this study. The ButCoA gene abundance was used as a proxy for high or low intestinal ButCoA levels over a longer period of time.^14^ Concentrations of the ButCoA gene can be used as a measure for intestinal acetate and butyrate availability.^19^ High ButCoA gene abundances correspond to high intestinal acetate and butyrate concentrations.^16^^,^^17^ Clinical and biochemical characteristics of the subjects per group (Table 1) showed no differences in baseline demographics between the two groups. No important differences in medication use were found between the groups. Subjects used the following medication: proton pump inhibitors (1 subject in the low and 3 in the high ButCoA group), oral anticoagulants (1 subject in the low and 2 in the high acetate/butyrate group), oral contraceptives (1 subject in the low and 2 in the high ButCoA group), antihypertensive medication (3 subjects in the low and 1 in the high ButCoA group), and statins (1 subject in the high ButCoA group). There were also no differences between groups in fecal SCFA levels, VAS hunger score, or mean daily caloric intake or consumption of carbohydrates, fat, protein, or dietary fiber (Table 1). GLP-1 differences were tested by comparing the 2-h area under the curve (AUC) (mean 905.90 and 954.05 mmol/L x min in the low and high ButCoA group, respectively, p = 0.455). Ghrelin 1.5-h AUC did not significantly differ between groups (mean 845.7 and 864.8 pmol/L x min, p = 0.633). Since absolutely no trend toward significance was observed, and because of logistic and financial limitations, the ghrelin assay was not repeated for the entire study population.

**Table 1:** Baseline characteristics for the high and low ButCoA group (N = 60)

	Low ButCoA group (N = 30)	High ButCoA group (N = 30)	p*-value*
Age (years)	56.5 [50.0–60.0]	56.0 [45.5–60.75]	0.947
No. of females (%)	17 (56.67%)	18 (60.0%)	1.000
BMI (kg/m^2^)	24.39 ± 4.24	25.32 ± 4.38	0.405
Body fat (%)	26.33 ± 8.05	27.99 ± 10.20	0.486
HbA1c	36.83 ± 3.65	35.73 ± 5.71	0.378
Systolic blood pressure (mmHg)	119.12 ± 15.05	122.96 ± 17.11	0.359
Diastolic blood pressure (mmHg)	74.33 ± 9.09	76.86 ± 9.44	0.296
Heart rate (bpm)	60.84 ± 12.06	63.31 ± 8.87	0.370
Cholesterol (mmol/L)	4.74 ± 0.97	4.78 ± 0.92	0.846
HDLc (mmol/L) (men)	1.16 ± 0.39	1.16 ± 0.16	0.994
HDLc (mmol/L) (women)	1.39 ± 0.35	1.47 ± 0.28	0.459
LDLc (mmol/L)	2.88 ± 0.96	2.82 ± 0.82	0.816
TG (mmol/L)	0.75 ± 0.45	0.79 ± 0.40	0.710
RMR (kcal/day)	1503.95 ± 245.05	1536.43 ± 238.01	0.607
Energy intake (kcal/day)	1877 ± 407.7	1839 ± 435.6	0.736
Fat intake (g/day)	75.59 ± 21.3	75.27 ± 24.6	0.958
Protein intake (g/day)	77.26 ± 21.3	77.60 ± 18.5	0.949
Carbohydrate intake (g/day)	198.1 ± 44.3	186.98 ± 58.9	0.426
Sugar intake (g/day)	72.24 ± 24.9	75.42 ± 36.2	0.698
Fiber intake (g/day)	22.45 ± 6.8	20.80 ± 6.8	0.360
Medication use	9 (30.0%)	11 (36.67%)	0.784
ButCoA abundance per 16S gene copies	7.17 ± 7.93	367.07 ± 205.97	**2.7e-16**
Fecal total SCFA (μmol/g)	337.3 [191.3–451.6]	319.6 [217.9–427.8]	0.866
Fecal acetate (μmol/g)	207 [102.3–231.9]	181.14 [129.11–242.43]	0.912
Fecal butyrate (μmol/g)	44.432 [20.538–78.398]	40.724 [23.709–76.173]	0.820
Fecal propionate (μmol/g)	66.39 [49.78–112.47]	66.72 [40.70–86.10]	0.382
VAS hunger score fasted	6.0 [4.0–7.0]	4.0 [3.0–6.75]	0.192
VAS hunger score after SMMT	1.0 [1.0–2.0]	1.0 [1.0–2.0]	0.569
VAS hunger score after fMRI	7.0 [5.75–8.0]	5.0 [5.0–7.0]	0.123

Tested with unpaired t test or Mann-Whitney U test, based on Gaussian distribution. Pearson chi-squared test had been performed to test for differences in sex and medication use between both groups. Numerical values are expressed as means ± standard deviations or median [IQR] depending on Gaussian distribution. No differences in characteristics were found between the groups.

ButCoA = Butyryl-coenzyme A transferase; BMI = body mass index; bpm = beats per minute; VAS = visual analog scale, SMMT = standardized mixed meal test; fMRI = functional magnetic resonance imaging.

### Glucose and insulin responses differed between groups

**Figure 1:**
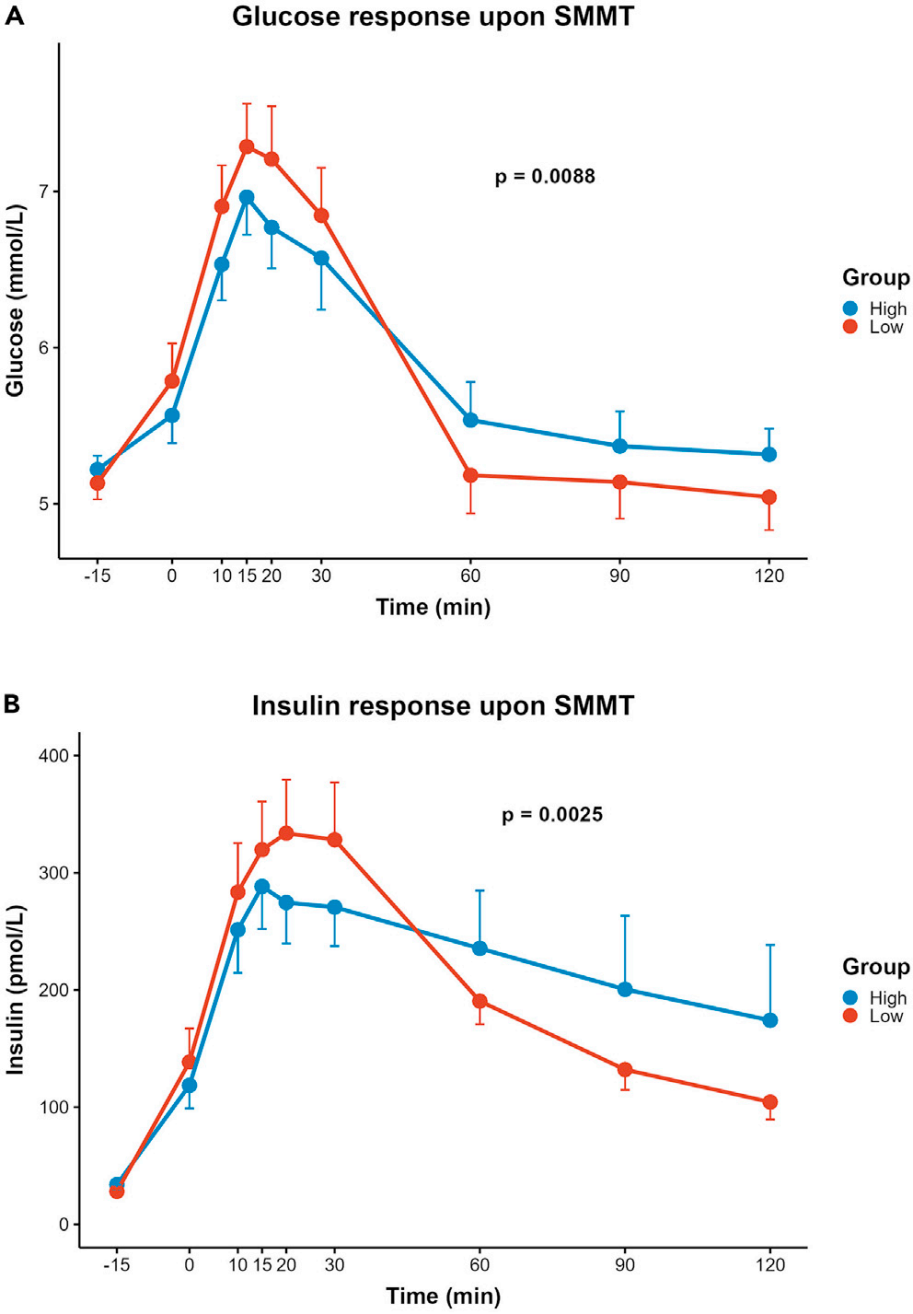
Glucose and insulin excursions upon mixed meal test (A) Glucose excursions upon mixed meal test. Glucose responses are depicted as mean ± S.E.M. Blue line shows the high ButCoA group, red line shows the low ButCoA group. There was no difference in plasma glucose level at baseline between the low and the high ButCoA group (5.13 ± 0.572 mmol/L vs. 5.22 ± 0.487 mmol/L). (B) Insulin excursions upon mixed meal test. Insulin responses are depicted as mean ± S.E.M. Blue line shows the high ButCoA group, red line shows the low ButCoA group. There was no difference in plasma insulin level at baseline between the low and the high ButCoA group (28.10 ± 15.24 pmol/L vs. 33.93 ± 29.32 pmol/L). mmol/L = millimole per liter; SMMT = standardized mixed meal test.

Fasting and postprandial glucose and insulin levels are depicted in Figure 1. There was no difference between the low and the high ButCoA group in insulin sensitivity estimated by Matsuda index, oral glucose insulin sensitivity (OGIS) index, or homeostatic model assessment for insulin resistance (HOMA-IR). Insulin secretion assessed by insulinogenic index did not differ between groups. Data are depicted in Table 2.

**Table 2:** Differences between the high and low ButCoA group in insulin sensitivity, insulin secretion, and incremental area under the curve (iAUC) of glucose and insulin and the respective ratios

Estimates of insulin sensitivity/secretion	Low ButCoA group (N = 30)	High ButCoA group (N = 30)	p*-value*
Fasted plasma glucose (mmol/L)	5.13 ± 0.572	5.22 ± 0.487	0.530
Fasted plasma insulin (pmol/L)	28.10 ± 15.24	33.93 ± 29.32	0.341

**Insulin sensitivity**			

Matsuda index	8.2 (4.7–10.6)	9.6 (5.4–11.8)	0.786
2-h OGIS index	298.8 (196.4–387.1)	312.7 (237.1–423.6)	0.916
Homeostatic model assessment for insulin resistance (HOMA-IR)	0.94 (0.43–1.12)	1.12 (0.7–1.3)	0.495

**Insulin secretion**			

Insulinogenic index (pmol/mmol)	110.5 (44.75–257.75)	83.5 (28.00–138.25)	0.387
Homeostatic model assessment for beta cell function (HOMA-β)	304.0 (199.1–508.7)	324.8 (249.5–380.6)	0.712

Oral disposition index (DI)	1.37 (0.489–2.94)	0.861 (0.615–1.44)	0.267
**Incremental area under the curve data**			

iAUC_glucose_	693.4 (627.7–785.0)	667.5 (606.3–778.2)	0.924
iAUC_insulin_	21161 (16175–29812)	19999 (18063–25482)	0.719
60-min iAUC_glucose_	394.4 (338.3–423.1)	372.2 (333.6–421.1)	0.561
60-min iAUC_insulin_	14156 (9139–17997)	12386 (11260–18007)	0.763
iAUC_insulin_/iAUC_glucose_ ratio (pmol/mmol)	30.88 (24.35–36.64)	30.50 (27.23–37.17)	0.924
60-min iAUC_insulin_/iAUC_glucose_ (pmol/mmol)	33.48 (28.71–49.99)	35.91 (27.43–44.09)	0.877

Values are expressed as means ± standard deviations or medians with interquartile range; differences were assessed using Mann-Whitney U test. No differences were found between both groups.

ButCoA = Butyryl-coenzyme A transferase; OGIS = oral glucose insulin sensitivity; HOMA-IR = homeostatic model assessment for insulin resistance; HOMA-β = homeostatic model assessment for beta cell function; DI = disposition index; iAUC = incremental area under the curve.

However, the two groups differed significantly in glucose and insulin curves over time during the 2-h period after the meal. The high ButCoA group showed lower plasma glucose and insulin values during the first 60 minutes after the SMMT, but higher levels during the second 60 minutes compared to the low group (Figure 1) (linear mixed model p < 0.01). This difference in postprandial glucose and insulin levels between high and low acetate producers persisted when analyzing only the overweight participants (BMI >25.0) (p = 0.045 and p = 0.0005, respectively), but was not observed when analyzing only the lean participants (p = 0.184 and p = 0.793).

Finally, the difference persisted upon exclusion of participants using medication affecting gastrointestinal motility (4 in the low and 4 in the high ButCoA group, respectively) (p = 0.01 and p = 0.006, respectively).

### No differences between groups in CNS responses while viewing food pictures

**Figure 2:**
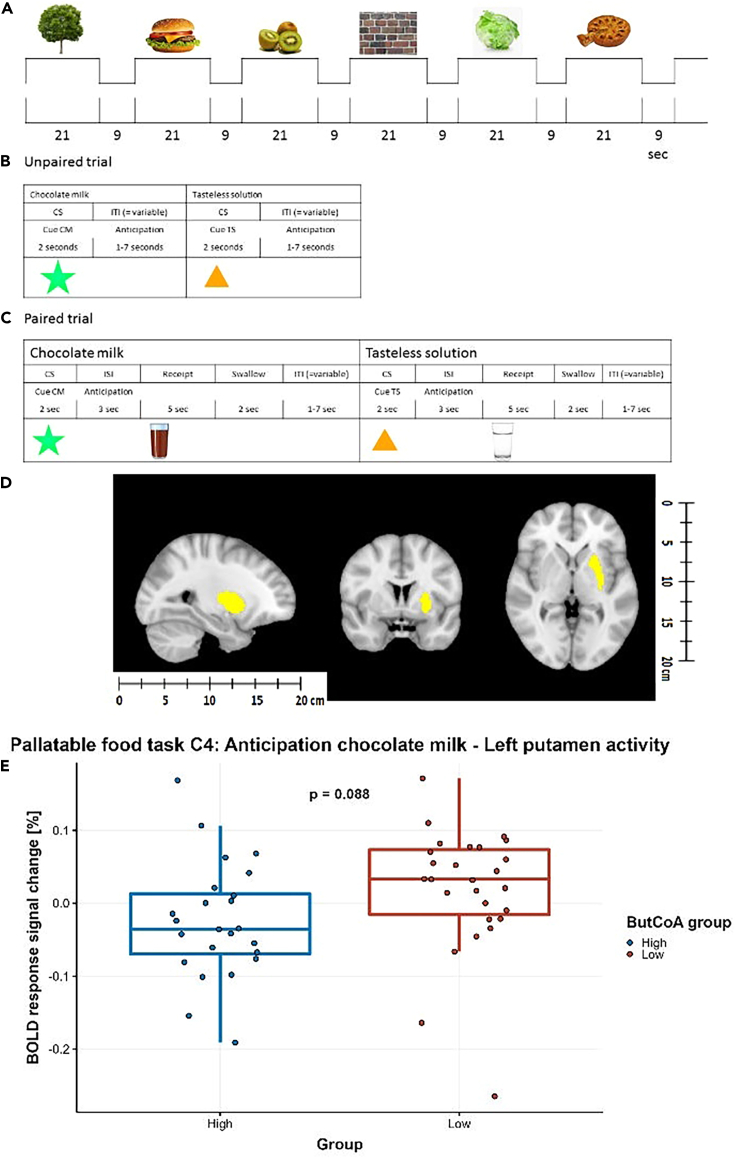
fMRI paradigm and outcome (A) Example of timing of picture blocks presentation during the food picture fMRI paradigm. (B) Timing of cue presentation during the chocolate milk fMRI paradigm in unpaired trials. (C) Timing of cue presentation and stimuli delivery during the chocolate milk fMRI paradigm in paired trials. (D) Anatomical mask of left putamen. Images were derived from the Harvard-Oxford subcortical atlas. Probability masks were downloaded and changed into binary masks using the fslmaths command, all images were thresholded at 60%. (E) Group differences in percent response change of BOLD signal upon anticipation of the receipt of chocolate milk vs. tasteless solution between high and low ButCoA group upon exposure to palatable food stimuli with error bars representing standard error. CS = conditioned stimulus; CM = chocolate milk; ISI = inter-stimulus interval; ITI = inter-trial interval; BOLD = blood-oxygen-level-dependent.

On whole brain level, we found a significant main effect for contrast 1 (food vs. non-food images) in the right insula and left orbitofrontal cortex (OFC) regions across both groups (Figure 2). Additionally, we found a main effect for contrast 2 (high-caloric vs. non-food images) in the bilateral insula and OFC (Table S1; Figure S2). However, no significant between-group differences were found in the predetermined ROIs for the viewing of food pictures.

### Lower left putamen activation in anticipation of food receipt in high ButCoA group compared to low ButCoA group

No main effects were observed on whole brain level across groups. When performing group-comparisons in the selected ROIs, during the anticipation of chocolate milk vs. tasteless solution, the activity in the left putamen tended to be lower in the high acetate group, compared to the low ButCoA group (respectively -0.035[-0.069 – 0.013]% and 0.033[-0.016 – 0.074]% blood-oxygen-level-dependent (BOLD) signal change, Bonferroni adjusted p value = 0.088) (Figure 2E). Furthermore, no significant left putamen activity was found in any of the groups when compared to 0% BOLD signal change in a one-sample t test.

### Fecal microbiota composition and beta diversity differ between high and low ButCoA group

#### Microbiota alpha and beta diversity in relation to intestinal acetate availability

**Figure 3:**
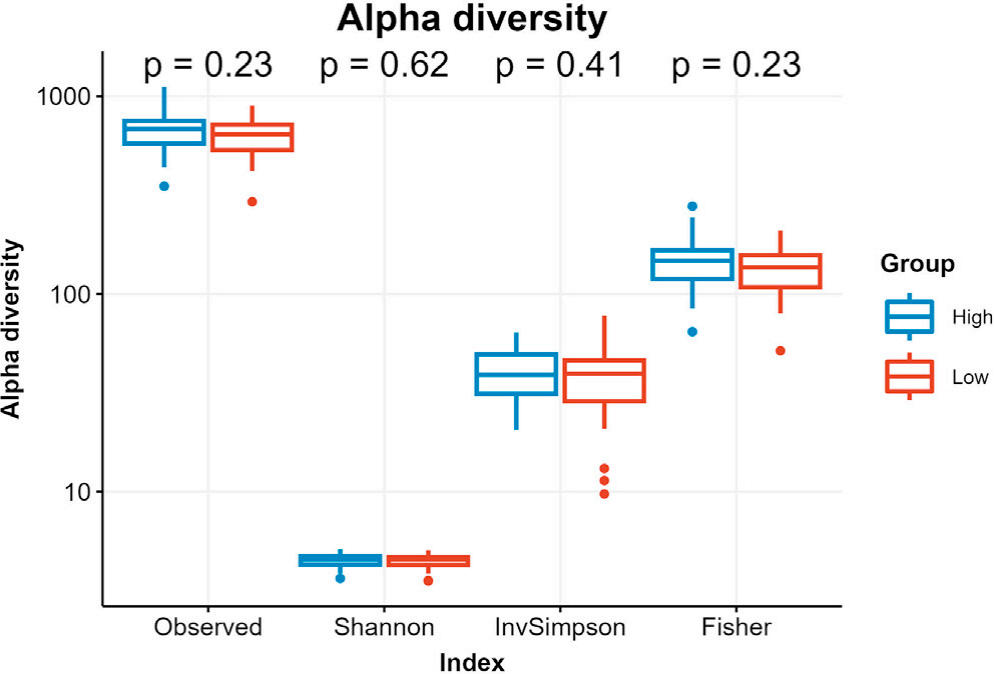
Alpha diversity per ButCoA group Tested as total number of observed taxa, Shannon index, Inverse Simpson index or Fisher’s alpha.

16S rRNA amplicon sequencing was performed on microbial DNA from fecal samples collected upon inclusion in the HELIUS study. There was no difference in alpha diversity between the groups (Figure 3). However, beta diversity was significantly different between the groups when adjusting for age, sex, and BMI, both in Weighted UniFrac distance (PERMANOVA p = 0.0031, R2 = 6.80%, explained variance by first 2 PCo 39.6% and 11.7%) (Figure S3) and in Bray-Curtis dissimilarity (PERMANOVA p = 0.0029, R2 = 3.11%, explained variance by first 2 PCo 11.7% and 7.6%).

#### Fecal microbiota composition in relation to intestinal acetate availability

**Figure 4:**
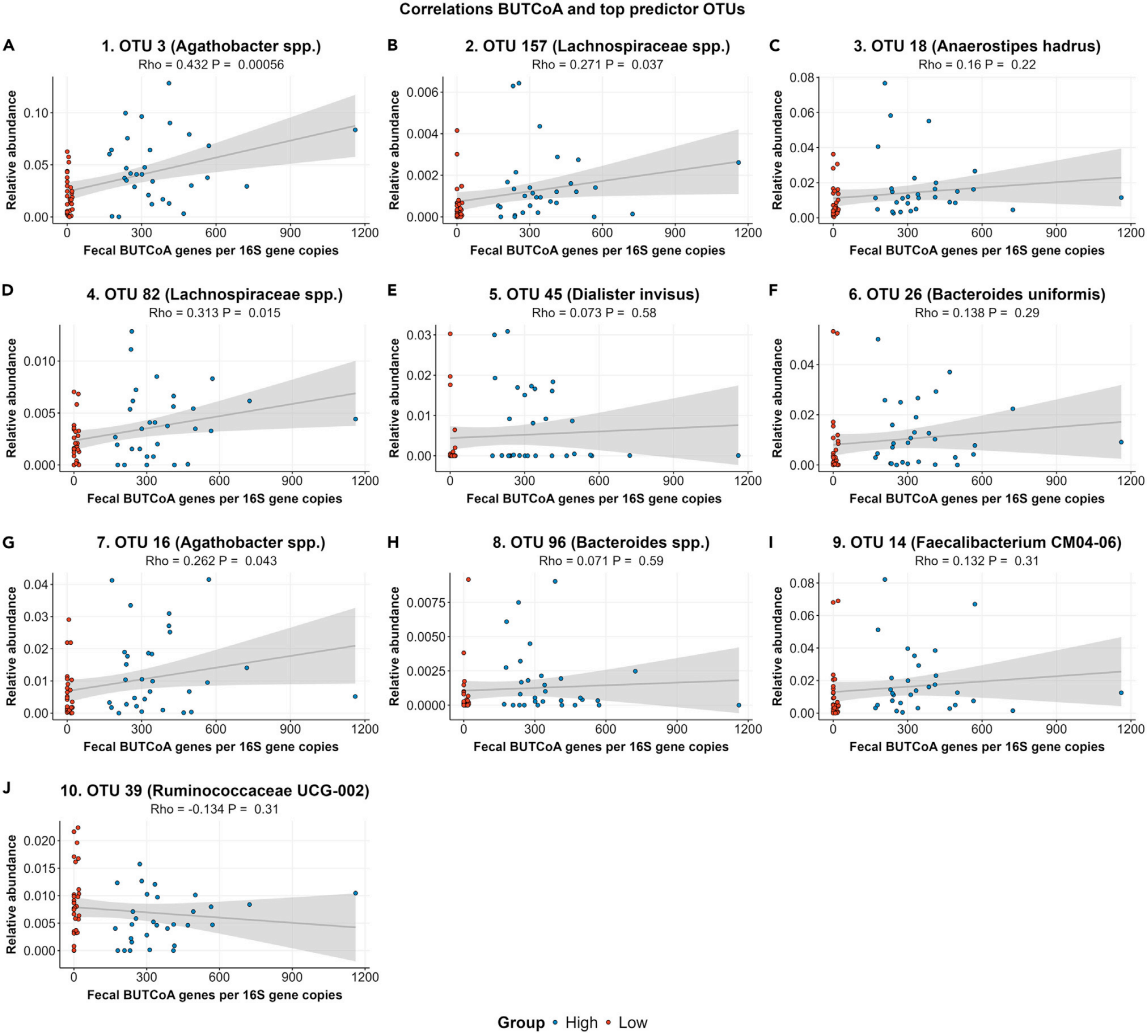
Correlation between ButCoA gene copies and ASVs (A) Agathobacter spp. (B) Lachnospiraceae spp. (C) Anaerostipes hadrus. (D) Lachnospiraceae spp. (E) Dialister invisus. (F) Bacteroides uniformis. (G) Agathobacter spp. (H) Bacteroides spp. (I) Faecalibacterium CM04-06. (J) Ruminococcaceae UCG-002. Selected ASVs were found by the machine learning classification algorithm with the levels of ButCoA gene copies. spp. = subspecies; OTU = operational taxonomic unit.

Using univariate analyses, no specific differences in amplicon sequence variants (ASVs) were observed between the low and the high intestinal ButCoA group, after false discovery rate correction for multiple comparisons. When applying an XGBoost machine learning classification model predicting whether a subject belonged to the low or high group, an AUC of 0.667 was obtained, only remotely higher than the permuted model, which had an AUC of 0.625, indicating a low predictive power of the actual model. Nevertheless, the true model median compared to all permuted outcome iterations differed significantly (p = 6.29e−07). Furthermore, no corresponding predictor ASVs were found between the real and the permuted model. The top ASVs with the highest feature importance for this model signified: *Agathobacter* spp. (previously known as the *Eubacterium rectale* group), Lachnospiraceae spp., *Anaerostipes hadrus*, and *Dialister invisus* (see Figures S1 and S4 for complete results). Of the top 10 predictor ASVs, the following significantly correlated to intestinal ButCoA concentrations: Agathobacter spp. and Lachnospiraceae spp. (Figure 4).

## Discussion

Here, we report a study in humans to investigate the association of intestinal acetate and butyrate availability with insulin sensitivity and insulin secretion as well as neural activity in reward and satiety-related areas. Insulin sensitivity and insulin secretion were similar, but glucose and insulin responses upon a standard high fiber meal were significantly lower in the high ButCoA group during the first hour and higher during the second postprandial hour.

Interventional studies in animals suggested that oral or colonic acetate or butyrate administration may improve insulin sensitivity and induce satiety.^6^^,^^7^ This was supported by a subgroup analysis of a preliminary study using oral butyrate supplementation.^12^ However, we did not find a relationship between intestinal acetate and butyrate availability (as determined by fecal ButCoA gene abundance) with insulin sensitivity or secretion in humans. Nevertheless, the high ButCoA group showed significantly different glucose and insulin responses upon a standard high fiber meal (p < 0.01). The high ButCoA group had lower glucose and insulin levels in the first hour, yet higher levels in the second hour after the mixed meal. In other words, both glucose and insulin had lower excursions in individuals with high intestinal acetate and butyrate availability. These data suggest a delayed glucose adsorption from the small intestine. Our results are in line with a preliminary study investigating the effect of vinegar on postprandial insulin and glucose in healthy humans.^20^ Vinegar consists largely of acetate and has a significant effect on postprandial glucose and insulin response with lower postprandial glycemic and insulinogenic responses in the first hour, but higher values in the second hour. A potential explanation could be the effects of intestinal acetate (as the conjugate base of acetic acid) binding to FFAR2/3 receptors on phasic and tonic gut motility. Additionally, higher SCFA availability in the upper gastrointestinal tract could exert an indirect effect on gastric emptying by stimulating satietogenic hormone secretion such as PYY and GLP-1, which also inhibit gastric motility.^21^ Delayed gastric emptying induced by GLP-1 receptor agonists^19^ resulted in postprandial glucose and insulin responses that are similar to those observed in our study.

We also observed that anticipating the receipt of chocolate milk vs. tasteless solution invoked a lower CNS response in the left putamen in the high ButCoA group compared to the low group, but this did not remain significant after correction for multiple comparisons. We also did not find a main effect for the anticipation of chocolate milk within the putamen or elsewhere in the brain within either group, suggesting that the task did not elicit the desired effect across groups. However, as the main effect includes both groups, it is also conceivable that the contrast elicits opposite effects in two subgroups, or a blunted effect in one of the groups, such that a main effect is not significant. Previous studies demonstrated that obesity is associated with higher responsiveness to palatable food images^22^ or cues that predict palatable food receipt.^23^ The putamen is part of the basal ganglia and is implicated in reward processing and conditioning and has previously been found to play a complex integrating role in food-related reward signals to behavior.^24^

Current research indicates that gut microbial-derived SCFAs might be able to affect brain physiology, both through direct and indirect interactions. Firstly, acetate and butyrate bind to FFAR2/3 receptors on enteric nerve cells, hereby inducing secretion of anorectic hormones such as GLP-1 and PYY.^25^ Furthermore, some evidence suggests acetate acts directly on hypothalamic centers of appetite control, but can also suppress appetite via the vagal-brainstem-hypothalamic pathway.^26^ In our current study, we did not administer exogenous (labeled) acetate and therefore we cannot define the exact mechanism of action. However, several studies have shown that higher peripheral levels of acetate correlate with higher CNS concentrations.^5^^,^^10^ For butyrate, central effects have not been shown in humans and are less plausible, since it reaches the periphery in considerably lower concentrations and there is limited evidence for it to cross the blood-brain barrier in significant amounts. However, indirect central effects of butyrate may be plausible.

Our results indicate that both glucose and insulin in individuals with high long-term intestinal acetate and butyrate availability had lower rates of change compared to the low group. Contrary to previous animal studies,^5^^,^^27^ we did not observe any relation between intestinal SCFA turnover and insulin sensitivity or secretion. However, our findings suggest a delay in gastric emptying or intestinal transit time. Prospective cohort studies should investigate the association of ButCoA gene amount with gastric emptying, intestinal absorption, and glucose metabolism. Future studies should also investigate whether acute or long-term systemic acetate and butyrate administration in humans affects glucometabolic health.

### Limitations of the study

First, fecal bacterial species abundancy and SCFA availability may only partially correlate with those in the small intestine,^28^ as we did not collect duodenal biopsies due to the invasive nature of this procedure. However, most of the SCFA adsorption takes place in the colon, of which the microbiota composition does correlate well to fecal matter.^14^ Second, despite the eminent difference in ButCoA levels, we did not observe any differences in fecal SCFA levels between the groups. It has to be noted, though, that these are known to be volatile, highly varying within subjects over time, and correspond poorly to intestinal adsorption rate.^28^ We therefore selected ButCoA abundance as a more stable measure of intestinal fermenting activity. Third, it could be suggested that the differences between the groups may be explained by confounding factors. Nevertheless, potential confounding factors including age, sex, BMI, medication use, resting metabolic rate, and energy intake did not differ between the two groups, suggesting that the metabolic differences between groups are in fact driven by intestinal metabolic variability, in particular acetate and butyrate availability. Fourth, it is currently not clear through which mechanism of action the SCFA exerts their effect. Gastric emptying has not been measured in our study. Future studies should therefore also include measures of gastric emptying when assessing the effects of SCFA.

## STAR★Methods

### Key resources table

**Table:** 

REAGENT or RESOURCE	SOURCE	IDENTIFIER
**Biological samples**		

Fecal samples	This study	N/A
Plasma samples	This study	N/A

**Critical commercial assays**		

QIAquick PCR Purification Kit	QIAGEN, Germany	N/A
Real-Time PCR System	Bio-Rad Laboratories, Hercules, CA, USA	CFX96™
GLP-1 ELISA kits	Northern Lights, Mercodia, Sweden	N/A
acylated ghrelin enzyme-linked immunoassay (ELISA) kits	BioVendor, Czech Republic	N/A
MiSeq system (RTA version 1.17.28, bundled with MCS version 2.5	Illumina	N/A
Cobas 8000 c702 analyzer	Roche, Basel, Switzerland	
ADVIA Centaur XP Immunoassay System	Siemens, Erlangen, Germany	N/A

**Deposited data**		

16S rRNA seq data	HELIUS cohort	EGAD00001004106

**Software and algorithms**		

USEARCH v11.0.667_i86linux64	Edgar et al.^29^	https://www.drive5.com/usearch/
fMRIPrep (v20.0.6)	Esteban et al.^30^	https://fmriprep.org/en/stable/
R Studio (v4.0.3)	N/A	N/A
phyloseq (v1.28.0)	R package	https://joey711.github.io/phyloseq/
dada2 (v1.12.1)	R package	https://www.bioconductor.org/
SILVA (v132)	Reference database	https://www.arb-silva.de/
MAFFT (v7.427)	Windows alignment program	https://mafft.cbrc.jp/
FastTree (v2.1.11 Double Precision)	Microbes Online	http://www.microbesonline.org/fasttree/
FSL (v6.0.0)	FMRIB, Oxford, UK	https://fsl.fmrib.ox.ac.uk/fsl/fslwiki
vegan (v2.5.6)	R package	https://cran.r-project.org/web/packages/vegan/index.html
ape (v5.4)	R package	https://cran.r-project.org/web/packages/ape/index.html
XGBoost (v0.9)	Python package	https://xgboost.readthedocs.io/en/stable/
numpy (v1.16.4)	Python package	https://numpy.org/
pandas (v0.25.1)	Python package	https://pandas.pydata.org/
scikit-learn (v0.21.2)	Python package	https://scikit-learn.org/stable/

**Other**		

3.0 Tesla Elition scanner	Philips Healthcare, Best, The Netherlands	N/A
Primary code in R language	GitHub - mwijdeveld	https://github.com/mwijdeveld/APRAISE/blob/main/RScript

### Resource availability

#### Lead contact

Further information and requests for resources and reagents should be directed to and will be fulfilled by the lead contact, Madelief Wijdeveld (madeliefwijdeveld@gmail.com).

#### Materials availability

Primers and PCR and ELISA kits were obtained from the commercial sources described in the STAR Methods key resources table. This study did not generate new unique reagents.

#### Data and code availability

• The 16S rRNA gene amplicon raw sequence data and associated metadata have been deposited at the European Genome-phenome Archive under accession code EGAD00001004106 (https://ega-archive.org/datasets/EGAD00001004106). The HELIUS data are all owned by the Amsterdam UMC, location AMC in Amsterdam, the Netherlands. Any researcher can request the data by submitting a proposal as outlined at http://www.heliusstudy.nl/.
• Primary code is available on the following github repository: https://github.com/mwijdeveld/APRAISE/blob/main/RScript.
• Any additional information required to reanalyze the data reported in this paper is available from the lead contact upon request.

### Experimental model and subject details

#### Study participants

From The Healthy Life in an Urban Setting (HELIUS) cohort in Amsterdam, a total of 60 subjects were included for this trial.^15^ In total, all 439 fecal samples of Caucasian participants who donated a stool sample at inclusion were selected and qPCR was performed to measure the concentration of the ButCoA gene, as a measure for intestinal acetate and butyrate availability, with high ButCoA gene amount corresponding to high intestinal acetate and butyrate concentrations^14^^,^^16^^,^^17^^,^^28^ (see section glucose and insulin responses differed between groups for further detail). All study protocols for this study were approved by the Ethical Review Board of Academic Medical Center, Amsterdam, (approval code: NL65063.018.18), and all patients provided written informed consent. Ethnicity was based on self-identification by participants. The study protocol was approved by the medical ethics committee of the Amsterdam University Medical Centre and performed in accordance with the Helsinki Declaration (version 2013). The clinical trial was preregistered online at: https://trialregister.nl/trial/7131.

From the 439 samples, all subjects belonging to the highest 15% and to the lowest 15% of ButCoA concentration received an invitation by mail to participate in the study. Exclusion criteria were immunodeficiency, serious heart, liver, renal or neurological disease, malignancies, inflammatory bowel disease, diabetes mellitus type 1 or 2, pregnancy or breast feeding, MRI contra-indications, alcohol or drug abuse, current use of antibiotics up to 3 months prior to the study visit and unstable body weight (>5% reported change during the previous 3 months). All interested candidates were tested for eligibility, first by telephone screening, followed by a physical screening in case all eligibility criteria were met. During this screening visit, respondents received materials and instructions for collecting stool samples and their medical history was determined, as well as the use of medication. If all eligibility criteria were met, an appointment was scheduled for the study visit. In total, 60 subjects (30 from the highest 15% and 30 of the lowest 15% ButCoA concentration) were included. All participants were Caucasian males or females aged 24-65 years. All participants filled out an online nutritional diary (https://mijn.voedingscentrum.nl) during 7 days prior to the study visit. In addition, during the study visit, participants filled out visual analogue scale (VAS) hunger scores to assess subjective appetite.

### METHOD details

#### Fecal sample analysis

##### Bacterial ButCoA gene quantification

Gene abundance was assessed by qPCR targeting the ButCoA gene, as described earlier.^31^ Total abundance of the ButCoA gene was expressed as a fraction of 16S rRNA gene copies in the fecal DNA. A more detailed description of the ButCoA quantification assay is included in the Methods S1 section.

##### Sequencing of the 16S rRNA gene

All stool samples of Caucasian HELIUS subjects were shipped to the Wallenberg Laboratory (Sahlgrenska Academy at University of Gothenburg, Sweden) to determine the fecal microbiome composition. Total genomic DNA was extracted from 150 mg aliquots according to the repeated bead beating protocol, as previously described.^32^ Composition of fecal microbiota was profiled by sequencing the V4 region of the 16S rRNA gene on a MiSeq system (RTA version 1.17.28, bundled with MCS version 2.5; Illumina) with 515F and 806R primers designed for dual indexing and the V2 kit (2x250bp paired-end reads; Illumina). Complete sequencing protocol is reported in the Methods S1 section.

##### Bioinformatics pipeline for 16S rRNA gene amplicon sequencing data

USEARCH (v11.0.667_i86linux64) was used to process the raw sequencing reads.^29^ Further pipeline information is reported in Methods S1.

#### Standardized mixed meal test

All participants were subjected to a standardized mixed meal test. The test started with insertion of an intravenous catheter in a distal arm vein, after which a baseline blood sample was drawn in order to measure fasting glucose, HbA1c, lipids, metabolites and liver enzymes. After the baseline blood withdrawal, subjects immediately ingested a standardized, fiber enriched meal within 15 minutes. The meal consisted of two slices of whole-wheat bread with 30 grams of strawberry jam and 30 grams of cheese, 250 ml of semi-skimmed milk and 250 ml of tea. Finally, 22.5 grams of long-chain inulin was added to the meal. The meal contained 503.1 kcal, 81.5 g carbohydrates, 15.1 g fat and 23.5 g protein. Blood samples were withdrawn for post-prandial measurements at T = baseline, 0:00, 0:10, 0:15, 0:20, 0:30, 1:00, 1:30 and 2:00. The blood samples were stored at -80°C.

#### Biochemistry

Blood was collected in Vacutainer tubes containing heparin, silica particles or ethylenediaminetetraacetic acid (EDTA), centrifuged at 1500 x g (15 min, 4°C) and plasma was stored at -80°C until further analyses. Glucose was measured with a commercial assay on a Cobas 8000 c702 analyzer (Roche, Basel, Switzerland). Plasma insulin was determined using the ADVIA Centaur XP Immunoassay System (Siemens, Erlangen, Germany), according to the manufacturer’s protocol. It was hypothesized for the high ButCoA group to have higher insulin sensitivity and glycaemic control compared to the low ButCoA group. In order to measure acylated and desacyl ghrelin, 200 mg/ml 4-(2-Aminoethyl)benzenesulfonyl fluoride hydrochloride (AEBSF) was added to plasma collected in an EDTA vial. Hereafter, human acylated ghrelin enzyme-linked immunoassay (ELISA) kits (BioVendor, Czech Republic) were used for ghrelin measurements. Ghrelin was measured for a subset of participants of 18 participants (N = 10 from the ‘high ButCoA group’ and 8 from the ‘low ButCoA group’) at T = baseline, 0:30, 1:00 and 1:30. GLP-1 was measured using Plasma GLP-1 ELISA kits (Northern Lights, Mercodia, Sweden). This was done for all participants at T = baseline, 0:30, 1:00 and 2:00.

#### Postprandial glucose response, insulin secretion and insulin sensitivity

First, postprandial plasma insulin and glucose levels were measured during the mixed meal test, as is standard practice when investigating glucose metabolism.^33^^,^^34^ Hereafter, insulin sensitivity was assessed using the Matsuda index, oral glucose insulin sensitivity (OGIS) and the Homeostatic Model Assessment for Insulin Resistance (HOMA-IR) index. Additionally, insulin secretion was calculated by means of insulinogenic index (IGI), the Homeostatic Model Assessment for beta cell function (HOMA-β) (formula: fasting plasma insulin (μIU/mL) × 360 / (fasting plasma glucose (mg/dL) – 63) and the oral disposition index (DI) (formula: IGI/HOMA-IR).^35^ For both insulin and glucose, we also calculated the incremental area under the curve (iAUC) for the entire 120 minutes and the first 60 minutes and the ratio of insulin to glucose incremental areas under the curve for the entire 120 minutes and the first 60 minutes was compared between the groups.^36^

#### Imaging protocol

##### MRI acquisition

MRI data were acquired using a 3.0 Tesla Elition scanner (Philips Healthcare, Best, The Netherlands) with a 32-channel receive-only head coil. 3D-T1-weighted (T1w) structural imaging data were acquired using a magnetization prepared rapid acquisition gradient echo (MPRAGE) sequence. Hereafter, two functional MRI scans were obtained consisting of two separate, food-related tasks, as described previously in more detail^37^^,^^38^ and briefly described below. Functional MRI data were acquired using a T2∗-weighted gradient-echo (GE) echo-planar imaging (EPI) sequence.

##### Virtual food task

This paradigm consisted of a blocked design to assess brain activation associated with visual presentation of food items. The task was designed to invoke a neural craving response in the 8-hour fasted individuals, based on stimuli displaying images of high-caloric (HC) food, low-caloric (LC) food images, and non-food (N) images (Figure 2A), as described earlier.^37^^,^^38^

##### Palatable food task

This paradigm consisted of an event-related design to assess brain activation in anticipation and response to palatable food stimuli in fasted state. Chocolate milk was administered as a palatable food stimulus. A tasteless solution was used as a neutral stimulus, designed to mimic the natural taste of saliva.^1^ Each session consisted visual cues (either a green star or an orange triangle) that signaled the delivery of 0.4 ml of chocolate milk or tasteless solution respectively (Figure 2C).

##### MRI data processing

MRI data were preprocessed using fMRIPrep 20.0.6.^30^ Each T1w scan was normalized to MNI space. Preprocessing of functional data included motion correction (FLIRT), distortion correction (3dQwarp), followed by co-registration to the T1w image using FLIRT (FSL 6.0.0). Independent Component Analysis-based Automatic Removal Of Motion Artifacts (ICA-AROMA) was used to non-aggressively denoise the data. Hereafter, data were spatially smoothed (6 mm full-width half maximum (FWHM)) and high-pass filtered within FSL/FEAT v6.0.0 (FMRIB Software Library; University of Oxford, Oxford, United Kingdom) (Methods S1 for further detail). For each functional scan, framewise displacement (FD) was determined for each consecutive frame relative to frame 1 using fMRIPrep 1.2.3. Data from subjects with extreme motion (mean framewise displacement (FD) > 0.5mm or > 10% of frames exceeded a FD of 1mm) were excluded from further analyses, this amounted to three subjects for the virtual and six for the palatable food task.

##### Whole brain and region of interest analysis

For every subject separately (at the first level), two contrasts were assessed per task. For the virtual food task contrast 1) viewing food vs. non-food pictures and 2) viewing high-caloric vs. non-food pictures were assessed. For the palatable food task contrast 3) receiving chocolate milk vs. tasteless solution and 4) anticipating the receipt of chocolate milk vs. the receipt of tasteless solution were assessed. In addition to these contrasts, motion outliers (FD > 1mm) were added as regressors to the first level model. These first-level contrast images were entered as input for the higher-level analyses in order to establish a main effect (one-sample t-test) across groups to validate the effect of each task among all subjects. Whole brain analysis was performed, in which peaks of cluster-based activity were examined using FSL. Results were considered to be significant at p < 0.05, family-wise error corrected on cluster extent, maintaining a Z threshold of 3.1. Hereafter, in order to test our primary hypothesis anatomical masks containing both unilateral and bilateral ROIs were used to visualize brain activation in our *a priori* anatomical ROIs only. Based on previous studies,^1^ we selected the amygdala, caudate nucleus, insula, orbitofrontal cortex (OFC) and putamen as our *a priori* regions of interest (ROIs). Part of the basal ganglia, the caudate nucleus and putamen are involved in reward processing and have previously been found to play an integrating role in food-related reward signals to behavior.^24^ The amygdala is the primary brain region regulating appetite and assigning emotion to food-related stimuli.^39^ The insula is part of a neural circuit involved in the prevention of overeating and ending feeding upon satiation.^40^ Furthermore, all ROIs are related to food reward and motivation.^1^^,^^41^ A Bonferroni correction for 8 multiple comparisons (2 contrasts in 4 regions) resulted into an adjusted alpha level of 0.00625.

### Quantification and statistical analyses

Unless stated otherwise, R Studio version 4.0.3 was used to perform all statistical analyses described below.

#### Power calculation

Based on previous studies investigating differences in acetate and butyrate levels,^42^^,^^43^ to detect a 25% reduction in iAUC postprandial glucose between the high and low acetate group (2-sided T-test with a desired alpha of 0.05 and a desired power of 0.8), a group sample size of 27 was necessary. In addition, to detect an anticipated difference in postprandial plasma insulin secretion of 10% (SD 10%), a minimum of 21 participants per group would be required. With regard to the secondary outcomes including fMRI, formal sample size calculations are less straightforward. Based on the results of previous fMRI studies performed by our group^37^^,^^38^ and others,^44^ addressing activity in comparable CNS circuits involved in satiety and reward regulation, we estimated that 27 participants per group rendered sufficient power. This is in line with studies of the statistical properties of a large fMRI cohort, that found that the sensitivity and reproducibility of group analyses reaches a plateau at N=27.^45^ We included 30 subjects per group, taking a 10% dropout into account.

#### Clinical data analysis

Differences in clinical characteristics, including insulin sensitivity and secretion, were tested with unpaired T-test or Mann-Whitney U test, depending on Gaussian distribution. To test for normal distribution and equality of variance, the Shapiro-Wilk Test and the Levene’s Test were used, respectively. Pearson Chi-Square test was used to test for differences in sex and medication use. A p-value < 0.05 was considered significant. All differences in clinical characteristics are summarized in Table 1; all differences in insulin sensitivity and secretion are summarized in Table 2. Numerical values are expressed as means ± standard deviations or median [IQR] depending on Gaussian distribution. N represents number of participants per group (high vs low ButCoA).

Changes in MMT-measured glucose and insulin were examined using linear mixed models (LMMs) using the nlme package (v3.1.147) with time (modelled as a natural cubic spline with 3 degrees of freedom) and acetate group as fixed effects and subject number as a random effect, reporting the significance of the interaction between the time and group effects. For ordinal scale outcomes (i.e. hunger and satiety) LMMs were implemented using the GLMMadaptive package (v0.6.8). All other statistical analyses and visualizations were implemented in R (v4.0.3) (R Core Team, 2016) using the tidyverse (v1.3.0) and ggplot2 package (v3.3.1). Differences in MMT-measured glucose and insulin are depicted in Figure 1. Both the glucose and insulin curve were plotted with spline interpolation in order to mimic the physiological postprandial plasma response more closely. Vertical bars represent standard error.

##### Fecal microbiome composition analyses

The vegan package (v2.5.6) was used to calculate alpha-diversity metrics (Shannon index, inverse Simpson index, and amplicon sequence variant (ASV) richness) (depicted in Figure 3) and Bray-Curtis dissimilarities. Weighted-Unifrac distances were calculated using the phyloseq package. Principal coordinate Analyses (PCoA) were performed using the ape package (v5.4). Weighted Unifrac distances are depicted in Figure S3. Permutational multivariate analysis of variance (PERMANOVA) was done using the adonis function from the vegan package using 1000 permutations. Each PERMANOVA was performed once without covariates and once adjusting for age, sex, and BMI.

#### Machine learning model

An eXtreme Gradient Boosting (XGBoost) Machine Learning algorithm of gradient boosted trees was used to generate a classification model predicting whether a subject belonged to the low or high intestinal ButCoA group. See Methods S1 for a detailed description of the model. Spearman rank correlation coefficients were calculated and averaged between all iterations for the top 10 predictor ASVs found by the model with Benjamini–Hochberg corrected p-values for multiple comparisons. Relative importance of the top 10 predictor ASVs is depicted in Figure S4. Here, relative importance of the top predictor ASV is set to 100%. Relative bacterial abundance of the top 10 predictor ASVs is depicted in Figure S1.

### Additional resources

This clinical trial was preregistered online at: https://trialregister.nl/trial/7131.

## Supplemental information

Document S1. Figures S1–S4, Table S1, Data S1, and Method S1
