# Supplementary material for: Intestinal acetate and butyrate availability is associated with glucose metabolism in healthy individuals
Source: iScience. 2023 Nov 16;26(12):108478. doi: 10.1016/j.isci.2023.108478 (PMC10716539; doi:10.1016/j.isci.2023.108478)
Supplement: Document S1. Figures S1–S4, Table S1, Data S1, and Method S1 [file mmc1.pdf]

iScience, Volume *26*

Supplemental information

Intestinal acetate and butyrate availability is associated with glucose metabolism in healthy individuals

Madelief Wijdeveld, Anouk Schrantee, Anouk Hagemeijer, Aart J. Nederveen, Torsten P.M. Scheithauer, Johannes H.M. Levels, Andrei Prodan, Willem M. de Vos, Max Nieuwdorp, and Richard G. Ijzerman

SUPPLEMENTARY DATA S1/METHODS S1

Additional information regarding fecal sample analysis and imaging protocol and results.

1. **Supplementary data S1**
2. **Supplementary Table S1**
3. **Supplementary methods S1**
4. **Supplementary Figure S1**
5. **Supplementary Figure S2**
6. **Supplementary Figure S3**
7. **Supplementary Figure S4**
8. **Supplementary reference list**

SUPPLEMENTARY DATA S1

1.1 Fecal microbiota composition in relation to intestinal acetate and butyrate availability

There was no difference in alpha-diversity between the groups. However, beta-diversity was significantly different between the groups when adjusting for age, sex and BMI, both in Weighted Unifrac distance (PERMANOVA p = 0.0031, R2 = 6.80%, explained variance by first 2 PCo 39.6% and 11.7%) (Fig. S3) and in Bray-Curtis dissimilarity (PERMANOVA p = 0.0029, R2 = 3.11%, explained variance by first 2 PCo 11.7% and 7.6%). Using univariate analyses, no specific differences in ASVs were observed between the low and the high intestinal ButCoA group, after FDR correction for multiple comparisons. When applying an XGBoost machine learning classification model predicting whether a subject belonged to the low or high group, an AUC of 0.667 was obtained, only remotely higher than the permuted model, which had an AUC of 0.625, indicating a low predictive power of the actual model. Nevertheless, the true model median compared to all permuted outcome iterations differed significantly (p = 6.29e−07). Furthermore, no corresponding predictor ASVs were found between the real and the permuted model. The top ASVs with the highest feature importance for this model signified: *Agathobacter* spp. (previously known as the *Eubacterium rectale* group), Lachnospiraceae spp., *Anaerostipes hadrus*, and *Dialister invisus* (see Fig. S1 for complete results).

Supplementary Table S1 Main effects of virtual food task in primary ROIs, related to Figure 2.

	Side			MNI			
		Z-max	Cluster size	x	y	z	*P* FWE corrected
Contrast 1: Food vs. non-food pictures							
Insula	R	5.21	131	38	6	-12	0.0269
OFC	L	5.4	239	-24	36	-14	1.05×10^{-3}
Contrast 2: High-caloric vs. non-food pictures							
Insula	L	5.17	833	-38	4	-10	2.97×10^{-9}
Insula	R	5.81	492	38	4	-10	2.38×10^{-6}
OFC	R	4.51	136	22	30	-14	0.0229

Montreal Neurological Institute (MNI) coordinates of peak voxels activated in a priori anatomical ROIs in the total group of participants with threshold P < 0.05 FWE whole brain corrected. For the BOLD activation during food vs. non-food pictures and high-caloric food vs. non-food pictures the main effects within in priori ROIs are presented. No main effects in primary ROIs were found for any of the palatable food task. The areas with significant differences are listed, including the cluster size of this effect, the Z value and the FWE corrected p-value. The last column describes the coordinates of the peak voxel of the observed difference in MNI space. ROIs = regions of interest; MNI = Montreal Neurological Institute; FWE = family-wise error; OFC = orbitofrontal cortex; L = left; R = right.

SUPPLEMENTARY METHODS S1

2.1 Fecal sample analysis

Bacterial acetate and butyrate availability. The gene abundance of ButCoA was measured to estimate bacterial acetate and butyrate availability. ButCoA transferase is essential for butyryl-CoA conversion into butyrate, a process in which acetate is used as an acceptor, high levels of ButCoA therefore signify high intestinal acetate levels, as well as butyrate levels. An in vitro study showed that various bacterial species show upregulation of ButCoA concentrations after being treated with high concentrations of acetate, implying a causal relation between acetate concentration and ButCoA activity (1). Furthermore, as could be expected, butyrate production increased simultaneously upon higher acetate concentrations added to the medium reservoir. Gene abundance was assessed by qPCR targeting the ButCoA gene. Per reaction, 10 ng of fecal DNA and 300 nM primers was used (2). Primers BCoATscrF (GCIGAICATTTCACITGGAAYWSITGGCAYATG) and BCoATscrR (CCTGCCTTTGCAATRTCIACRAANGC) were used for this qPCR protocol. The amplification cycle used was: 1 cycle of 95°C for 3 min; 40 cycles of 95°C, 53°C, and 72°C for 30 s each with data acquisition at 72°C; 1 cycle each of 95°C and 55°C for 1 min; and a stepwise increase of the temperature from 55 to 95°C (at 10 s/0.5°C) to obtain melting curve data. The gene abundance in copies/µL was estimated according to a standard, which was synthesized from gDNA of *Faecalibacterium prausnitzii* via PCR. The PCR product was purified with QIAquick PCR Purification Kit (Qiagen, Germany); DNA concentration measured with Nanodrop-1000 and copies per µL were calculated according to the DNA concentration as well as amplicon size. qPCR amplifications were performed in duplicate using CFX96™ Real-Time PCR System (Bio-Rad Laboratories, Hercules, CA, USA). Each reaction was amplified using Sensifast Sybr No Rox 2x kit (BIOLINE, UK). Similarly, the total copies of bacteria were assessed using EUBAC primers as previously described (3). The abundance of the ButCoA gene was determined by dividing the number of copies of the ButCoA gene by that of 16S rRNA gene copies in the fecal DNA. This was subsequently used to make an estimation of the rate of total intestinal acetate into butyrate conversion. Fecal SCFA concentrations (acetate, butyrate, and propionate) were determined in the collected stool samples using liquid chromatography-tandem mass spectrometry (LC-MS/MS) (4).

Sequencing of the 16S rRNA gene. Total genomic DNA was extracted from 150 mg aliquots according to the repeated bead beating protocol, as previously described (5). 16S rRNA genes from each sample were amplified in duplicate reactions in volumes of 25 µl containing 1x 5PRIME (HotMasterMix; 5PRIME), 200nM of each primer, 0.4 mg ml-1 BSA, 5% dimethylsulfoxide, and 20 ng of genomic DNA. PCR was carried out under the following conditions: initial denaturation for 3 min at 94°C; annealing for 60 seconds at 52°C; elongation for 90 seconds at 72 °C; and a final elongation step for 10 minutes at 72 °C. Duplicates were combined, purified with the Nucleospin Gel and PCR Clean-Up kit (Machery-Nagel), and quantified using the Quant-iT PicoGreen dsDNA kit (Invitrogen). Purified PCR products were diluted to 10 ng μl^{-1} and pooled in equal amounts. The pooled amplicons were purified again using Ampure magnetic purification beads (Agencourt) to remove short amplification products. Negative controls were included for each sample. The absence of detectable PCR products in these negative controls was confirmed with gel electrophoresis. Composition of fecal microbiota was profiled by sequencing the V4 region of the 16S rRNA gene on a MiSeq system (RTA version 1.17.28, bundled with MCS version 2.5; Illumina) with 515F and 806R primers designed for dual indexing and the V2 kit (2x250bp paired-end reads; Illumina).

Bioinformatics pipeline for 16S rRNA gene amplicon sequencing data. For paired-end merging, we used 30 maximum allowed differences in the overlapping region ("maxdiffs") for the merging step (using the "fastq_mergepairs" command) and maximum 1 expected error ("fastq_maxee") as a quality filter threshold (using the "fastq_filter" command). Expected error-based read quality filtering is described in detail elsewhere (6). After merging paired-end reads and quality filtering, remaining contigs were dereplicated and unique sequences were denoised using the UNOISE3 algorithm in order to obtain ASVs (7). All merged reads were subsequently mapped against the resulting ASVs to produce a count table. ASVs not matching expected amplicon length were filtered out (i.e. ASV sequences longer than 260 bp or shorter than 250 bp). Taxonomy was assigned with the 'assignTaxonomy' function from the 'dada2' R package (v1.12.1) (8) and the SILVA (v132) reference database. ASVs sequences were then aligned using MAFFT (v7.427) (9) using the auto settings. A phylogenetic tree was constructed from the resulting multiple sequence alignments with FastTree (v2.1.11 Double Precision) (10) using a generalized time-reversible model ('-gtr'). The AVS table, taxonomy and tree were integrated using the 'phyloseq' R package (v1.28.0) (11). The ASV table was rarefied to 14932 counts per sample.

2.2 Imaging protocol

Anatomical imaging data acquisition and preprocessing. 3D-T1-weighted (T1w) structural imaging data were acquired with the following scan parameters: repetition time/echo time = 7/3.19 ms; flip angle = 9°; 180 sequential slices, field of view 256 x 240 x 180 mm, resolution 1 x 1 x 1 mm. The T1-weighed (T1w) images were corrected for intensity non-uniformity (INU) using N4BiasFieldCorrection (12) (Advanced Normalization Tools (ANTs) 2.2.0), and used as T1w-reference throughout the workflow. The T1w-reference was then skull-stripped using antsBrainExtraction.sh (ANTs 2.2.0), using OASIS as target template. Spatial normalization to the ICBM 152 Nonlinear Asymmetrical template version 2009c (13) was performed through nonlinear registration with antsRegistration (ANTs 2.2.0) (14), using brain-extracted versions of both T1w volume and template. Brain tissue segmentation of cerebrospinal fluid (CSF), white-matter (WM) and gray-matter (GM) was performed on the brain-extracted T1w using fast (FSL 6.0.0) (15).

Functional imaging data acquisition and preprocessing. Functional MRI data were acquired with the following scan parameters: repetition time/echo time = 1500/30 ms; flip angle 73°, field of view 240 x 240 x 131.75 mm, resolution 2.5 x 2.5 x 2.5 mm, slice gap = 0.25 mm, multiband factor 2, SENSE factor 2, 400 volumes for the virtual food protocol, 820 volumes for the palatable food protocol. For each BOLD run (across all tasks and sessions), the following preprocessing was performed for each subject separately. First, a reference volume and its skull-stripped version were generated using a custom methodology of fMRIPrep. A deformation field to correct for susceptibility distortions was estimated based on two echo-planar imaging (EPI) references with opposing phase-encoding directions, using 3dQwarp (16) (AFNI 20160207). Based on the estimated susceptibility distortion, an unwarped BOLD reference was calculated for a more accurate co-registration with the anatomical reference. The BOLD reference was then co-registered to the T1w reference using FLIRT (FSL 5.0.9) (17) with the boundary-based registration (18) cost-function. Co-registration was configured with nine degrees of freedom to account for distortions remaining in the BOLD reference. Head-motion parameters with respect to the BOLD reference (transformation matrices, and six corresponding rotation and translation parameters) are estimated before any spatiotemporal filtering using MCFLIRT (FSL 6.0.0) (19). The BOLD time-series (including slice-timing correction was applied) were resampled onto their original, native space by applying a single, composite transform to correct for head-motion and susceptibility distortions. These

resampled BOLD time-series will be referred to as *preprocessed BOLD in original space*, or just *preprocessed BOLD.* First, a reference volume and its skull-stripped version were generated using a custom methodology of fMRIPrep. Automatic removal of motion artifacts using independent component analysis (ICA-AROMA) (20) was performed on the *preprocessed BOLD on MNI space* time-series after removal of non-steady state volumes and spatial smoothing with an isotropic, Gaussian kernel of 6mm FWHM (full-width half-maximum). Corresponding "non-aggressively" denoised runs were produced after such smoothing. Additionally, the "aggressive" noise-regressors were collected and placed in the corresponding confounds file. The BOLD time-series were resampled to *MNI152NLin2009cAsym* standard space, generating a *preprocessed BOLD run in MNI152NLin2009cAsym space*. First, a reference volume and its skull-stripped version were generated using a custom methodology of fMRIPrep. Several confounding time-series were calculated based on the *preprocessed BOLD*: framewise displacement (FD), DVARS and three region-wise global signals. FD and DVARS are calculated for each functional run, both using their implementations in *Nipype* (21). The three global signals are extracted within the CSF, the WM and the whole-brain masks. Additionally, a set of physiological regressors were extracted to allow for component-based noise correction (*CompCor*) (22). Principal components are estimated after high-pass filtering the *preprocessed BOLD* time-series (using a discrete cosine filter with 128 s cut-off) for the two *CompCor* variants: temporal (tCompCor) and anatomical (aCompCor). Six tCompCor components are then calculated from the top 5% variable voxels within a mask covering the subcortical regions. This subcortical mask is obtained by heavily eroding the brain mask, which ensures it does not include cortical GM regions. For aCompCor, six components are calculated within the intersection of the aforementioned mask and the union of CSF and WM masks calculated in T1w space, after their projection to the native space of each functional run (using the inverse BOLD-to-T1w transformation). The head-motion estimates calculated in the correction step were also placed within the corresponding confounds file. All resampling can be performed with a single interpolation step by composing all the pertinent transformations (i.e. head-motion transform matrices, susceptibility distortion correction when available, and co-registrations to anatomical and template spaces). Gridded (volumetric) resamplings were performed using antsApplyTransforms (ANTs), configured with interpolation to minimize the smoothing effects of other kernels (23). Non-gridded (surface) resamplings were performed using mri_vol2surf (FreeSurfer).
Many internal operations of fMRIPrep use *Nilearn* 0.4.2 (24), mostly within the functional processing workflow.

Virtual food task paradigm. All pictures were presented via E-prime 2.0 (Psychology Software Tools, Inc., Pittsburg, PA). The stimuli were presented in 18 separate blocks: 6 blocks of stimuli displaying images of high-caloric (HC) food and 6 blocks of low-caloric (LC) food images, and 6 blocks of non-food (N) images (Fig. 1a), serving as control stimuli (25, 26). Within each block 7 pictures were presented for 2.5 seconds each, separated by a 0.5 second blank screen. All blocks were alternated with a 9 s grey screen with a fixation cross. For all subjects, the order of blocks was exactly as follows: LC, N, LC, HC, N, HC, N, LC, HC, N, HC, LC, N, HC, LC, HC, LC, N.

Palatable food task paradigm. Chocolate milk (Chocomel© (FrieslandCampina); 73 kcal, 1.6 g fat, 11 g sugar per 100 ml) was administered as a palatable food stimulus via a Vygon Lectroflex V-Green extension line that was placed in the participants' mouth before start of the scan, which was connected to a Infusomat® Space P pump (B Braun Medical Inc. Pennsylvania, USA) outside of the scan room programmed to deliver 0.4 ml of fluid at a time. A tasteless solution was used as a neutral stimulus,

designed to mimic the natural taste of saliva (consisting of 2.5 mM $NaHCO_3$ and 25 mM KCl) (26-28) and administered via the same oral route. Each session consisted of 100 trials, during which a visual cue was presented (either a green star or an orange triangle) that signaled the delivery of 0.4 ml of chocolate milk or tasteless solution respectively (Fig. 1c) (26, 28). Images were presented for 2 s in random order, followed by a 3 s blank screen with a fixation cross (anticipation period) and 2 s of stimulus delivery, after which the participants were instructed to swallow. In 40% of the trials, however, the cue was not followed by a stimulus delivery in order to maintain an unconditioned response (Fig. 1b).

Region of interest analysis. Anatomical masks containing both unilateral and bilateral ROIs were used to perform ROI analyses. The masks were created using the Harvard-Oxford cortical (insula) and subcortical (amygdala, caudate and putamen) atlases thresholded at 60%. Consequently, for each contrast, the mean signal intensity of every single participant was extracted per anatomical region for each contrast separately using Featquery. Parameter estimates were converted to percent signal change. For each group, parameter estimates were entered into a one sample t-test in order to assess whether the signal change was significantly different from zero. Hereafter, the group outcomes were compared using a two-sample t-test. Since we performed eight tests for the ROI analysis (4 ROI analyses for both left and right hemisphere), adjusted alpha level was set at 0.00625. Finally, to examine differences on whole-brain level outside of our primary ROIs, the first level contrast images were entered as input for a two-sample t-test for each contrast separately to establish a group effect. The latter was performed as an exploratory analysis to identify possible activation pattern differences between the groups for the different tasks in all regions of the brain. We hypothesized for ButCoA to have a beneficial effect on appetite suppression, and therefore for the high ButCoA group to show lower craving response upon food anticipation in reward-related areas, but a higher reward response upon food receipt.

2.3 Statistical analyses

Power calculation. Based on previous studies investigating differences in acetate and butyrate levels (29, 30), a group sample size of 27 was necessary to detect a 25% reduction in insulin sensitivity between the high and low ButCoA group (2-sided T-test with a desired alpha of 0.05 and a desired power of 0.8). In addition, to detect an anticipated difference in postprandial plasma insulin secretion of 10% (SD 10%), a minimum of 21 participants per group would be required. With regard to the secondary outcomes including fMRI, formal sample size calculations are less straightforward. Based on the results of previous fMRI studies performed by our group (25, 26) and others (31), addressing activity in comparable CNS circuits involved in satiety and reward regulation, we estimated that 27 participants per group rendered sufficient power. This is in line with studies of the statistical properties of a large fMRI cohort, that found that the sensitivity and reproducibility of group analyses reaches a plateau at N=27 (32). We included 30 subjects per group, taking a 10% dropout into account.

Fecal microbiome composition analyses. The vegan package (v2.5.6) was used to calculate alpha-diversity metrics (Shannon index, inverse Simpson index, and amplicon sequence variant (ASV) richness) and Bray-Curtis dissimilarities. Weighted-Unifrac distances were calculated using the phyloseq package. Principal coordinate Analyses (PCoA) were performed using the ape package (v5.4). Permutational multivariate analysis of variance (PERMANOVA) was done using the adonis function from the vegan package using 1000 permutations.

Machine learning model. An eXtreme Gradient Boosting (XGBoost) Machine Learning algorithm of gradient boosted trees was used to generate a classification model predicting whether a subject belonged to the low or high intestinal ButCoA group. Machine learning was implemented in Python (v. 3.8.5) using the XGBoost (v. 0.90), numpy (v. 1.16.4), pandas (v. 0.25.1) and scikit-learn (v. 0.21.2) packages. Gradient boosted tree models were used in a nested cross-validation structure to prevent overfitting and ensure robustness of results. The model was built using an iterative flow and consisted of 200 iterations, in each iteration, the dataset was randomly split into an 80% training set and a 20% test set. Thereafter, five-fold cross-validation was performed within the training set in order to fit and optimize the model hyperparameters. Two random variables were added to the predictor data during each iteration to serve as a benchmark. Finally, the optimized model was then tested on the examples in the test set. The ranked feature importance list were recorded for each iteration. Spearman rank correlation coefficients were calculated and averaged between all iterations for the top 10 predictor ASVs found by the model with Benjamini–Hochberg corrected p-values for multiple comparisons.

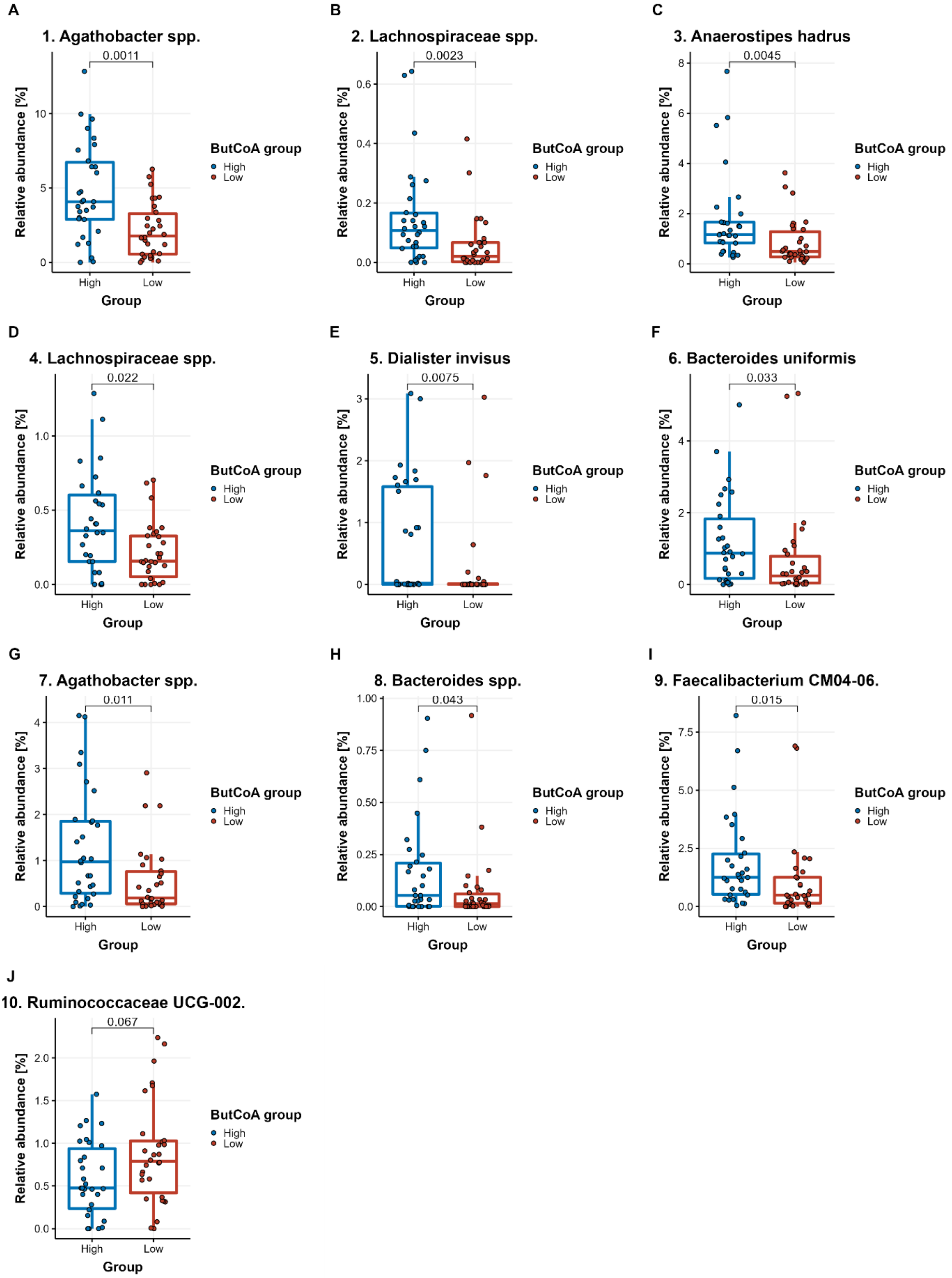

Supplementary Figure S1 Relative bacterial abundance of top 10 predictor ASVs per group, related to Figure 4. The plots feature the median (center line), upper and lower quartiles (box limits) and relative abundance per subject (points).

a Main effect contrast 1: Food vs. non-food

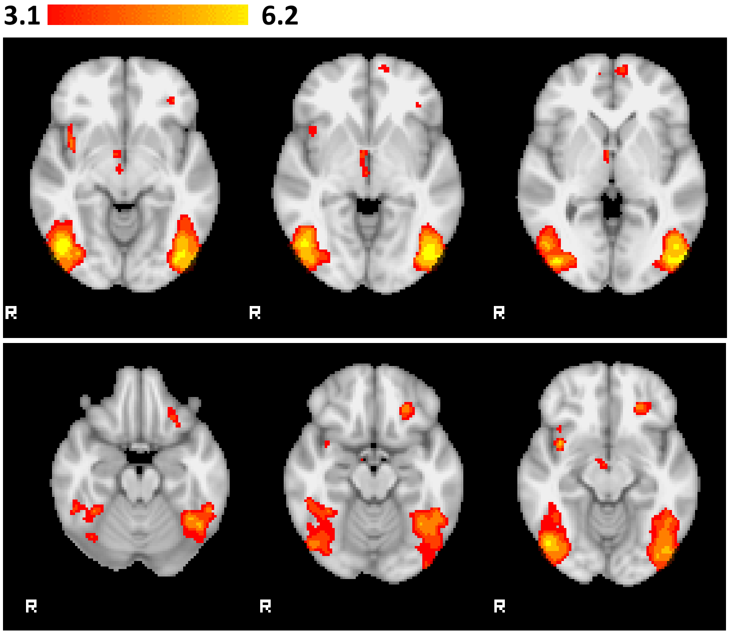

b Main effect contrast 2: High-caloric vs. non-food

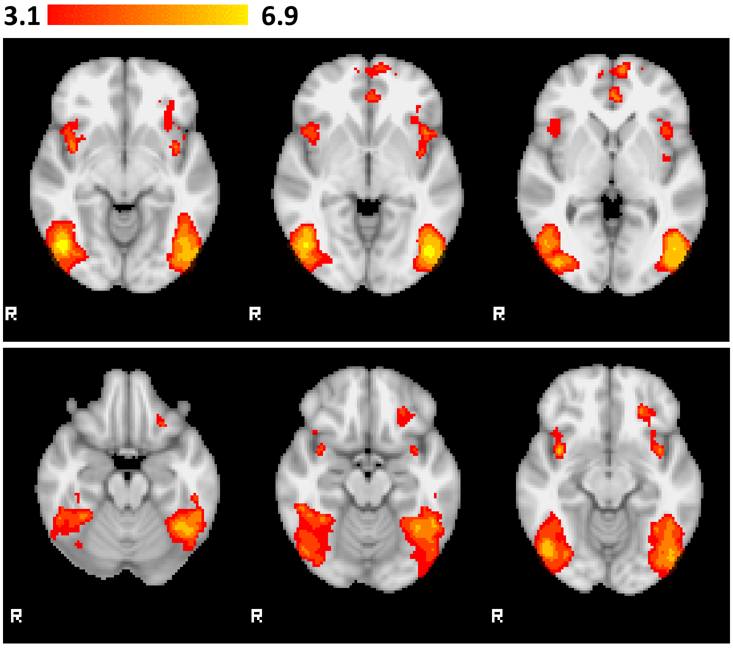

Supplementary Figure S2 Voxel-based activation on whole-brain level, related to Figure 2.
Effects are tested across all participants (main effect) with $P < 0.05$ FWE correction for the contrasts (a) viewing food vs. non-food pictures and (b) viewing high-caloric vs. non-food pictures. Color bar represents Z-value.

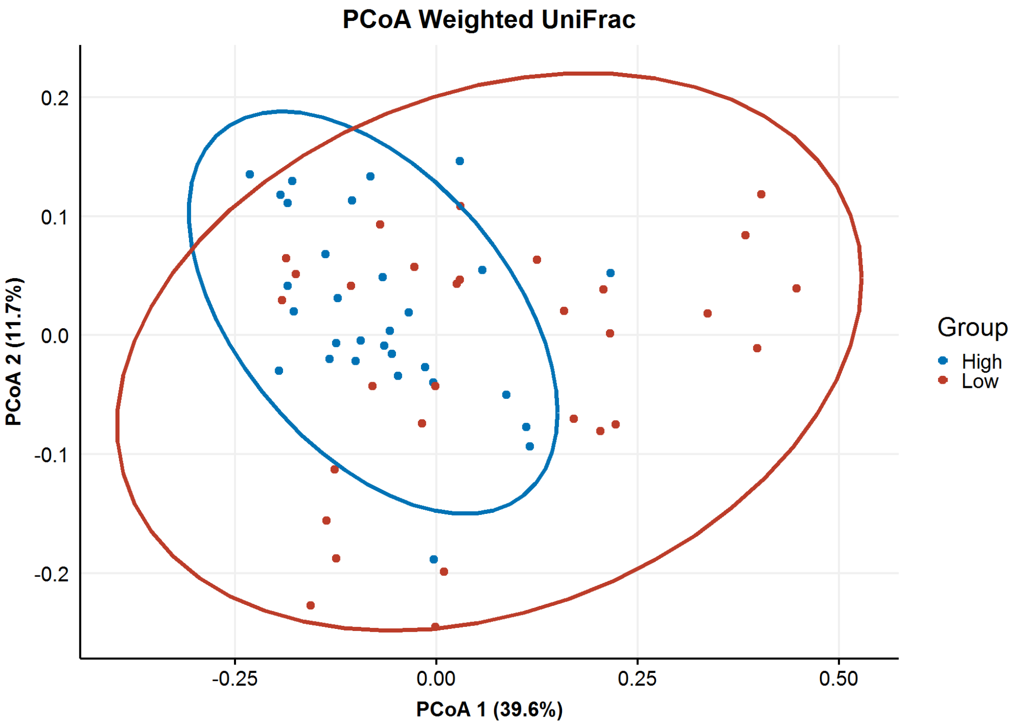

Supplementary Figure S3 Principal coordinate analysis (PCoA) on beta-diversity (Unweighted UniFrac) between low and high intestinal ButCoA group, related to Figure 4.

PERMANOVA: p = 0.0031, R2 = 6.80%, explained variance by first 2 PCo 39.6% and 11.7%. Each point represents one sample from one participant (blue: high acetate group, red: low acetate group). Closeness of points represents similarity of microbial composition.

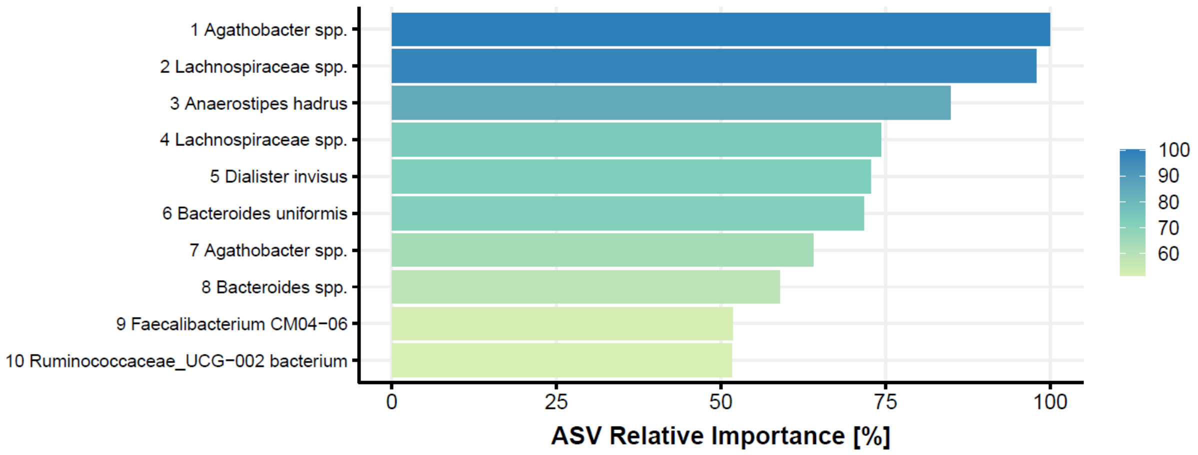

Supplementary Figure S4 Relative feature importance of top 10 predictor ASVs from gut microbiota composition for ButCoA group (high vs. low), related to Figure 4.

Relative importance is determined with respect to the most important predictor of the model, which was set to 100%. Predictor ASVs were identified applying an XGBoost classification model.

Figure legends

Supplementary Table S1: Main effects of virtual food task in primary ROIs, related to Figure 2.

Montreal Neurological Institute (MNI) coordinates of peak voxels activated in a priori anatomical ROIs in the total group of participants with threshold $P < 0.05$ FWE whole brain corrected. For the BOLD activation during food vs. non-food pictures and high-caloric food vs. non-food pictures the main effects within in priori ROIs are presented. No main effects in primary ROIs were found for any of the palatable food task. The areas with significant differences are listed, including the cluster size of this effect, the Z value and the FWE corrected p-value. The last column describes the coordinates of the peak voxel of the observed difference in MNI space.

Supplementary Figure S1: Relative bacterial abundance of top 10 predictor ASVs per group, related to Figure 4.

The plots feature the median (center line), upper and lower quartiles (box limits) and relative abundance per subject (points).

Supplementary Figure S2: Voxel-based activation on whole-brain level, related to Figure 2.

Effects are tested across all participants (main effect) with $P < 0.05$ FWE correction for the contrasts (a) viewing food vs. non-food pictures and (b) viewing high-caloric vs. non-food pictures. Color bar represents Z-value.

Supplementary Figure S3: Principal coordinate analysis (PCoA) on beta-diversity (Unweighted UniFrac) between low and high intestinal ButCoA group, related to Figure 4.

PERMANOVA: $p = 0.0031$, $R2 = 6.80\%$, explained variance by first 2 PCo 39.6% and 11.7%. Each point represents one sample from one participant (blue: high acetate group, red: low acetate group). Closeness of points represents similarity of microbial composition.

Supplementary Figure S4: Relative feature importance of top 10 predictor ASVs from gut microbiota composition for ButCoA group (high vs. low), related to Figure 4.

Relative importance is determined with respect to the most important predictor of the model, which was set to 100%. Predictor ASVs were identified applying an XGBoost classification model.

SUPPLEMENTARY REFERENCES

1. Diez-Gonzalez, F., Bond, D.R., Jennings, E., and Russell, J.B. (1999). Alternative schemes of butyrate production in Butyrivibrio fibrisolvens and their relationship to acetate utilization, lactate production, and phylogeny. Archives of microbiology 171(5), 324-30. DOI:10.1007/s002030050717.

2. Louis, P., and Flint, H.J. (2007). Development of a Semiquantitative Degenerate Real-Time PCR-Based Assay for Estimation of Numbers of Butyryl-Coenzyme A (CoA) CoA Transferase Genes in Complex Bacterial Samples. Applied and Environmental Microbiology 73(6), 2009.

3. Nadkarni, M.A., Martin, F.E., Jacques, N.A., and Hunter, N. (2002). Determination of Bacterial Load by Real-Time PCR Using a Broad-Range (Universal) Probe and Primers Set. Microbiology 148, 257-66. DOI:10.1099/00221287-148-1-257.

4. Karlsson, F.H., Tremaroli, V., Nookaew, I., Bergström, G., Behre, C.J., Fagerberg, B., Nielsen, J., and Bäckhed, F. (2013). Gut metagenome in European women with normal, impaired and diabetic glucose control. Nature (London) 498(7452), 99-103. DOI:10.1038/nature12198.

5. Mobini, R., Tremaroli, V., Ståhlman, M., Karlsson, F., Levin, M., Ljungberg, M., Sohlin, M., Bertéus Forslund, H., Perkins, R., Bäckhed, F., and Jansson, P.A. (2017). Metabolic effects of Lactobacillus reuteri DSM 17938 in people with type 2 diabetes: A randomized controlled trial. Diabetes, Obesity and Metabolism 19(4), 579-89. DOI:10.1111/dom.12861.

6. Edgar, R.C., and Flyvbjerg, H. (2015). Error filtering, pair assembly and error correction for next-generation sequencing reads. Bioinformatics 31(21), 3467-82.

7. Edgar, R.C. (2016). UNOISE2: improved error-correction for Illumina 16S and ITS amplicon sequencing. BioRxiv, 081257.

8. Callahan, B.J., McMurdie, P.J., Rosen, M.J., Han, A.W., Johnson, A.J.A., and Holmes, S.P. (2016). DADA2: High-resolution sample inference from Illumina amplicon data. Nature Methods 13(7), 581-3.

9. Katoh, K., Misawa, K., Kuma, K., and Miyata, T. (2002). MAFFT: a novel method for rapid multiple sequence alignment based on fast Fourier transform. Nucleic Acids Research 30(14), 3059-66.

10. Price, M.N., Dehal, P.S., and Arkin, A.P. (2010). FastTree 2 – Approximately Maximum-Likelihood Trees for Large Alignments (FastTree 2). PLoS ONE 5(3), e9490. DOI:10.1371/journal.pone.0009490.

11. McMurdie, P.J., and Holmes, S. (2013). phyloseq: An R Package for Reproducible Interactive Analysis and Graphics of Microbiome Census Data.(Research Article). PLoS ONE 8(4), e61217. DOI:10.1371/journal.pone.0061217.

12. Tustison, N.J., Avants, B.B., Cook, P.A., Yuanjie, Z., Egan, A., Yushkevich, P.A., and Gee, J.C. (2010). N4ITK: Improved N3 Bias Correction. IEEE Transactions on Medical Imaging 29(6), 1310-20. DOI:10.1109/TMI.2010.2046908.

13. Fonov, V.S., Evans, A.C., McKinstry, R.C., Almli, C.R., and Collins, D.L. (2009). Unbiased nonlinear average age-appropriate brain templates from birth to adulthood. Neuroimage 47, S102-S. DOI:10.1016/S1053-8119(09)70884-5.

14. Avants, B.B., Epstein, C.L., Grossman, M., and Gee, J.C. (2008). Symmetric diffeomorphic image registration with cross-correlation: Evaluating automated labeling of elderly and neurodegenerative brain. Medical Image Analysis 12(1), 26-41. DOI:10.1016/j.media.2007.06.004.

15. Zhang, Y., Brady, M., and Smith, S. (2001). Segmentation of brain MR images through a hidden Markov random field model and the expectation-maximization algorithm. IEEE Transactions on Medical Imaging 20(1), 45-57. DOI:10.1109/42.906424.

16. Cox, R.W., and Hyde, J.S. (1997). Software tools for analysis and visualization of fMRI data. NMR in Biomedicine 10(4-5), 171-8. DOI:10.1002/(SICI)1099-1492(199706/08)10:4/5<171::AID-NBM453>3.0.CO;2-L.

17. Jenkinson, M., and Smith, S. (2001). A global optimisation method for robust affine registration of brain images. Medical Image Analysis 5(2), 143-56. DOI:10.1016/S1361-8415(01)00036-6.

18. Greve, D.N., and Fischl, B. (2009). Accurate and robust brain image alignment using boundary-based registration. Neuroimage 48(1), 63-72. DOI:10.1016/j.neuroimage.2009.06.060.

19. Jenkinson, M., Bannister, P., Brady, M., and Smith, S. (2002). Improved Optimization for the Robust and Accurate Linear Registration and Motion Correction of Brain Images. Neuroimage 17(2), 825-41. DOI:10.1006/nimg.2002.1132.

20. Pruim, R.H.R., Mennes, M., van Rooij, D., Llera, A., Buitelaar, J.K., and Beckmann, C.F. (2015). ICA-AROMA: A robust ICA-based strategy for removing motion artifacts from fMRI data. NeuroImage 112, 267. DOI:10.1016/j.neuroimage.2015.02.064.

21. Power, J.D., Mitra, A., Laumann, T.O., Snyder, A.Z., Schlaggar, B.L., and Petersen, S.E. (2014). Methods to detect, characterize, and remove motion artifact in resting state fMRI. NeuroImage 84(4), 320-41. DOI:10.1016/j.neuroimage.2013.08.048.

22. Behzadi, Y., Restom, K., Liau, J., and Liu, T.T. (2007). A component based noise correction method (CompCor) for BOLD and perfusion based fMRI. NeuroImage 37(1), 90-101. DOI:10.1016/j.neuroimage.2007.04.042.

23. Lanczos, C. (1964). Evaluation of Noisy Data. Journal of the Society for Industrial & Applied Mathematics, Series B: Numerical Analysis 1(1), 76-85. DOI:10.1137/0701007.

24. Abraham, A., Pedregosa, F., Eickenberg, M., Gervais, P., Muller, A., Kossaifi, J., Gramfort, A., Thirion, B., and Varoquaux, G. (2014). Machine Learning for Neuroimaging with Scikit-Learn. Frontiers in Neuroinformatics 8, 14.

25. ten Kulve, J., Veltman, D., Bloemendaal, L., Barkhof, F., Deacon, C., Holst, J., Konrad, R., Sloan, J., Drent, M., Diamant, M., and Ijzerman, R. (2015). Endogenous GLP-1 mediates postprandial reductions in activation in central reward and satiety areas in patients with type 2 diabetes. Diabetologia 58(12), 2688-98. DOI:10.1007/s00125-015-3754-x.

26. Doornweerd, S., De Geus, E., Barkhof, F., Van Bloemendaal, L., Boomsma, D., Van Dongen, J., Drent, M., Willemsen, G., Veltman, D., and Ijzerman, R. (2018). Brain reward responses to food stimuli among female monozygotic twins discordant for BMI. Brain Imaging and Behavior 12(3), 718-27. DOI:10.1007/s11682-017-9711-1.

27. Stice, E., Spoor, S., Bohon, C., and Small, D.M. (2008). Relation between obesity and blunted striatal response to food is moderated by TaqIA A1 allele. Science (New York, NY) 322(5900), 449. DOI:10.1126/science.1161550.

28. ten Kulve, J.S., Veltman, D.J., van Bloemendaal, L., Groot, P.F.C., Ruhé, H.G., Barkhof, F., Diamant, M., and Ijzerman, R.G. (2016). Endogenous GLP1 and GLP1 analogue alter CNS responses to palatable food consumption. Journal of endocrinology 229(1), 1-12. DOI:10.1530/JOE-15-0461.

29. Johansson Boll, E.V., Ekström, L.M.N.K., Courtin, C.M., Delcour, J.A., Nilsson, A.C., Björck, I.M.E., and Östman, E.M. (2015). Effects of wheat bran extract rich in arabinoxylan oligosaccharides and resistant starch on overnight glucose tolerance and markers of gut fermentation in healthy young adults. European journal of nutrition 55(4), 1661-70. DOI:10.1007/s00394-015-0985-z.

30. Sandberg, J.C., Björck, I.M.E., and Nilsson, A.C. (2016). Rye-Based Evening Meals Favorably Affected Glucose Regulation and Appetite Variables at the Following Breakfast; A Randomized Controlled Study in Healthy Subjects. PloS one 11(3), e0151985-e. DOI:10.1371/journal.pone.0151985.

31. Stoeckel, L.E., Weller, R.E., Cook, E.W., Twieg, D.B., Knowlton, R.C., and Cox, J.E. (2008). Widespread reward-system activation in obese women in response to pictures of high-calorie foods. NeuroImage (Orlando, Fla) 41(2), 636-47. DOI:10.1016/j.neuroimage.2008.02.031.

32. Thirion, B., Pinel, P., Tucholka, A., Roche, A., Ciuciu, P., Mangin, J.F., and Poline, J.B. (2007). Structural Analysis of fMRI Data Revisited: Improving the Sensitivity and Reliability of fMRI Group Studies. IEEE transactions on medical imaging 26(9), 1256-69. DOI:10.1109/TMI.2007.903226.
